# Preface

**DOI:** 10.1186/2047-783X-19-S1-I1

**Published:** 2014-06-19

**Authors:** Dieter Häussinger, Helmut Sies

**Affiliations:** 1 Clinic of Gastroenterology, Hepatology and Infectious Diseases, Heinrich Heine University, 40225 Düsseldorf, Germany; 2 Institute of Biochemistry and Molecular Biology I, Heinrich Heine University, 40225 Düsseldorf, Germany

## 

Here, we present the Proceedings of the I. International Conference of the Collaborative Research Center SFB 974 “Communication and Systems Relevance in Liver Damage and Regeneration” , held at Düsseldorf, Germany, November 15-16, 2013. The SFB 974 started its research work in 2012 following the SFB 575 “Experimental Hepatology” (2000 to 2011). The liver is long known for its high regenerative potential, and understanding of mechanisms underlying liver regeneration will be of great importance for future treatment of chronic liver diseases, which affect more than one million people in Germany.

Lectures were given by internationally renowned scientists and by members of the Collaborative Research Center SFB 974. Topics comprised the role of stem cells in liver regeneration, systems biology of liver regeneration, and the interplay between liver damage, regeneration and cancer development. Most importantly, young scientists of the SFB 974 presented their data on posters and in short oral contributions. Competent help by the Scientific Coordinator, Dr. Nora Luniak, is gratefully acknowledged.

We thank all authors who contributed to this publication.

Düsseldorf, April 2014

